# Upper Extremity Infection Related to Intravenous Drug Use: Considering the True Cost of the COVID-19 Pandemic and Lockdown

**DOI:** 10.1177/15589447221077377

**Published:** 2022-02-22

**Authors:** Yaeesh Sardiwalla, Omri Nachmani, Emma Price, Minh Huynh, Christopher Coroneos, Matthew McRae

**Affiliations:** 1 McMaster University, Hamilton, Ontario, Canada; 2 University of Toronto, Ontario, Canada

**Keywords:** infection, diagnosis, psychosocial, research and health outcomes, health policy, epidemiology, outcomes

## Abstract

**Background:**

The COVID-19 pandemic caused significant morbidity and mortality in people who inject drugs (PWID). Upper extremity soft tissue infections are frequently associated with intravenous drug use (IVDU) due to poor compliance with aseptic technique. In Canada, multiple safe injection sites providing clean injection supplies closed, leaving many PWID with no alternatives to inject safely. It was hypothesized that these closures will correspond with increased morbidity and mortality among PWID. The main objective of this study was to determine the effect of the COVID-19 pandemic on the incidence of upper extremity infections in PWID.

**Methods:**

This was a retrospective chart review study. The primary outcome of interest was the frequency of upper extremity infections in PWID. Data were filtered to include only those patients presenting to the emergency department between March to June of 2019 and 2020. Chi-squared analysis was used to compare the number of IVDU patients among patients with upper extremity skin infections between these time periods.

**Results:**

The number of IVDU patients treated for upper extremity infections in Hamilton significantly increased during the pandemic, relative risk = 2.0 (95% confidence interval [CI]: 1.3-2.9, *P* = .0012,) while total upper extremity infections numbers have decreased overall. During the pandemic, PWID made up a larger proportion of upper extremity infections (*χ*^2^ = 10.444, *P* = .00123). Demographic data such as age and sex of IVDU patients presenting with upper extremity infection was not significantly affected by the pandemic.

**Conclusions:**

The effect of the pandemic on accessing harm reduction services has led to evident increases in morbidity as described by this study. Further research on the impact of closures in PWID is needed to quantify these harms and work toward mitigation strategies.

## Introduction

Skin and soft tissue infections are the third most common emergency department (ED) diagnosis and the most common reason for hospital admission.^1-3^ Upper extremity soft tissue infections specifically are frequently associated with intravenous drug use (IVDU) due to poor compliance with aseptic technique including the common practice of sharing, reusing, or licking needles.^4-6^ With repeated injection into a single site, skin and deeper tissue are damaged, resulting in local ischemia and necrosis that increase susceptibility to infection.^7^ The most commonly isolated species are gram positive skin flora, including *Staphylococcus aureus* and *Streptococcus pyogenes*.^4,8^

Treatment of these soft tissue infections may be nonoperative, such as immobilization and antibiotics, or surgical debridement of necrotic tissue.^9^ A delay in diagnosis and treatment may lead to complications such as necrotizing fasciitis, myositis, bacteremia, and sepsis.^4,8,10^ Some believe ED visits and subsequent hospital admissions due to IVDU to be a financial strain on healthcare systems.^11,12^ For instance, a study conducted in 2001 at St. Paul’s Hospital in Vancouver found the fully allocated average cost to take care of patient with a history of IVDU cost an average of $610.33 per day (95% confidence interval [CI]: $575.70–$644.96).^11^

Safe injection facilities were first introduced to Canada in Vancouver in response to a high rate of HIV infection and fatal overdoses.^13^ These facilities offer people who inject drugs (PWID): nurse supervision to inject previously obtained drugs, sterile tools, access to emergency overdose responses, and referrals to health and social services.^13,14^ Currently, the COVID-19 pandemic has had unparalleled effects on the healthcare system, among which included changes to hours and services available at harm-reduction services including safe injection facilities.^15,16^ It was therefore hypothesized that PWID who lost access to a safe drug supply, clean equipment, and safe locations to use drugs has a corresponding increase in morbidity and mortality due to infections during the pandemic.^17-19^ The aim of this study is to investigate the changes in incidence of upper extremity infections related to IVDU during the COVID-19 pandemic.

## Materials and Methods

This was a retrospective chart review study. The main objective of this study was to determine the effect of the COVID-19 pandemic on the frequency of upper extremity infections for PWID. This study was approved by the Hamilton Integrated Research Ethics Board (HiREB) for retrospective data collection through chart review (#11297). The primary outcome of interest was the frequency of upper extremity infections for PWID. Secondary outcomes such as rates of hospitalizations, antibiotics prescribed and route, and need for surgery were investigated. De-identified ED data of upper extremity abscess, infection, or cellulitis was obtained through data requests to Hamilton Health Sciences (HHS) and St. Joeseph’s Hospital Hamilton. Data were filtered to include only those patients presenting to ED between 20 March 2019 to 20 June 2019 or 20 March 2020 to 20 June 2020. During March 2020, uncertainty of how COVID-19 spreads, testing backlog, and rapid increase in hospitalizations has led many nonessential services including safe injection sites to unilaterally close. By comparing these time periods, we aimed to capture changes in upper extremity soft tissue infections associated with the height of the COVID-19 pandemic. Patients were included in this study if they were 18 years or older and had a prior history of IV drug use as reported in the electronic medical records or previous ED presentations. The presentation characteristics and administered interventions were collected for all patients who met inclusion criteria. Presentation fields included: warmth, erythema, edema, fever, pain, fluctuance, hypotension, and tachycardia. Interventions of interest were antibiotics, route of antibiotics, interventions (irrigation and debridement or surgery), and admission.

**Figure 1.**
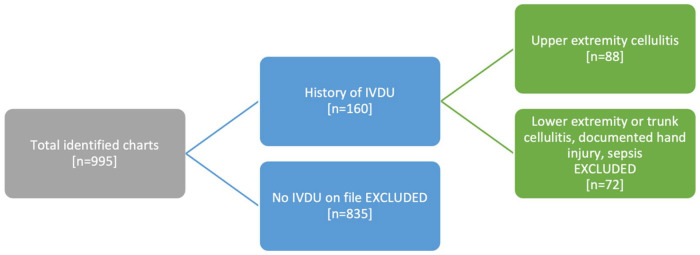
Flow diagram showing inclusion and exclusion of study participants based on chart review. *Note*. IVDU = intravenous drug use.

Patients were identified using ICD10 codes (Supplemental Appendix A). The initial data request yielded 569 incidents of upper extremity cellulitis presentations to the ED before the pandemic and 426 during the pandemic across all Hamilton sites in the specified dates. Data were then filtered to patients with upper extremity infections and prior history of or ED presentation related to drug abuse, opiate overdose, and polysubstance use, yielding 160 total encounters for the chart review stage. These charts were reviewed in detail to verify the inclusion criteria by 2 independent assessors (Figure 1). Excluded patients ranged from patients who did not inject drugs, had lower extremity infections, presented with sepsis, or had a traumatic injury as a precedent to their infection. Sepsis was excluded as it is not specific enough to skin infections. Statistical analysis of a chi-squared test was performed using R Statistical Software (R Foundation for Statistical Computing, Vienna, Austria).

## Results

For the date ranges included in the study, the total number of upper extremity infection seen in Hamilton Emergency Department was 569 in 2019 prepandemic and 426 in 2020 during the pandemic. The number of cases unrelated to IVDU decreased from 533 in 2019 to 373 in 2020. In contrast, while 36 (6.3%) IVDU presented to the ED for upper extremity infections in 2019, that number rose to 53 (12.4%) during the COVID-19 pandemic. This represents a statistically significant rise with a relative risk = 2.0 (95% CI: 1.3-2.9, *P* = .0012) while total upper extremity infections numbers have decreased overall. During the pandemic, PWID made up a larger proportion of upper extremity infections (*χ*^2^ = 10.444, *P* = .00123). Further a Poisson distribution analysis showed a 47% relative increase in cases of upper extremity infections in PWID between the time periods with normal assumptions for this test (95% CI: 0.96–2.25 and *P* = .073). There was a decrease in the proportion of females and an increase in average number of symptoms during COVID-19. However, due to the small sample size the demographic data of patients such as age and sex did not significantly change before and during the pandemic (*P* = .7113, *P* = .5119, respectively).

**Figure 2.**
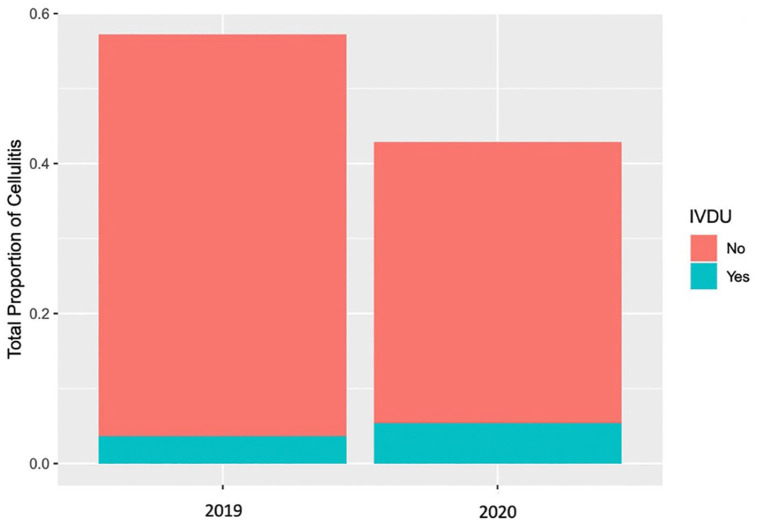
Proportion of upper extremity cellulitis attributable to IVDU and non-IVDU patient populations before and during the COVID-19 pandemic. *Note.* IVDU = intravenous drug use; COVID-19 = coronavirus disease.

On average, patients during the pandemic presented with similar number of clinical symptoms of infection before and during the pandemic. Table 1 summarizes the occurrences of upper extremity infections in IV drug users presenting to the ED before and during the COVID-19 pandemic and Figure 2 demonstrates this increase graphically.

**Table 1.** Upper Extremity Infections in IVDU Before and During the COVID-19 Pandemic.

Cases	2019	2020
Non-IVDU	533	373
IVDU	36	53
M	0.66	0.85
F	0.34	0.15
Median age	33	34
Avg symptoms	3.23	4.74

*Note.* IVDU = intravenous drug use; COVID-19 = coronavirus disease.

The treatment of upper extremity infections represents a surrogate market of severity. As some patients received multiple interventions, column sums do not reflect total infections for that year. Administration of oral/IV antibiotics and surgical drainage have not changed considerably between these two time points. Table 2 summarizes prescribed interventions in the IVDU patient population.

**Table 2.** Treatments Prescribed for IVDU During Each Time Period.

Treatment	2019	2020
Oral Antibiotics	22	39
Intravenous Antibiotics	15	15
Incision and Drainage	10	7

*Note.* IVDU = intravenous drug use.

## Discussion

Our observational study indicates a larger proportion of patients presenting with upper extremity infections had a history of IV drug use during the pandemic period (12%) compared to the pre-COVID-19 period (6%). This study does not identify changes in the severity of disease presentation or elucidate an identifiable reason as why there may have been an increase. It does, however, confirm the authors hypothesis that there has been an increase in cases within the ED during the COVID-19 pandemic.

The approximately 50% increase in the total number of cases during the pandemic would likely be atypical compared to normal annual variance in cases, but since a multiple year average and spread preceding the pandemic was not calculated, this could not be stated with statistical certainty. The aim of the study was to promote conversations based on the observed trends we have noted. The effect of the pandemic on accessing harm reduction services has been well described in other contexts, and our hope is that these data can promote a conversation on the potential harms being caused and further research into the impact closure of services has had.^17-19^

Vulnerable populations who are afflicted with unstable housing, addiction, and food insecurity have had a disproportionate negative impact with COVID-19 induced closures of services that aimed to address these issues. Poor communication regarding clear but safe rules to allow continued functioning of these services are partly to blame for this. Many healthcare services transferred the provision of their services digitally, but this was not often possible for harm reduction services due to the nature of the services provided and access issues for the populations served.^20,21^ Many organizations were forced to rely on SARS-era rules to govern their opening and cleaning policies, which rendered services inaccessible.^22,23^ Clear guidance and leadership are necessary in these continuing times of need as we enter the next stage of the pandemic. If governments and healthcare worked in a more collegial fashion with these organizations acknowledging their importance to population health, perhaps many patients in need would have been able to access these critical services.

The economic marginal benefit derived from harm reduction programs compared to health concerns of IVDU populations is well described in the literature.^24-27^ The marginal cost effectiveness for the previous of hepatitis C infection from preventing needle sharing alone has a value that ranges from $1705 to $97,203 per individual using the service depending on their age.^24^ Similarly, the benefit-cost ratio ranges from 5.9 to 20.6 and the cost-effectiveness value ranges from $1705 to $5969 (cost per lifetime treatment).^24^ It is also well reported that those with a history of substance use have a significant higher healthcare cost with one study demonstrating an increase in mean 6 year cost as much as $56,178 more than those without history of substance use.^25^ The IVDU also has a much higher healthcare utilization even when compared those using other substances like cocaine, sedatives, inhalants, and hallucinogens.^26^ There has also been a dramatic increase in costs of hospitalization with a previous Canadian study demonstrating the average cost of hospitalization in IVDU population was $24,982 CAD compared to the cost of a standard hospital stay was $7359 CAD.^27^

The economic savings of this become apparent when you realize that increased ED visits and associated costs to treating 72 extra upper extremity infections a year (our study showed 18 more in 3 months) could cost Hamilton’s hospitals upward of $44,000 if cost data from the St. Paul’s study is used.^11^ This figure understates the true cost when you recognize that more severe infection, hospitalization, and surgery all become more likely with increasing numbers of infection.

The study was able to clearly demonstrate that although general upper extremity infection presentation to the ED had decreased during the same three months prior to the pandemic, the incidence in infection in PWID had increased. By understanding the performance of the baseline population, statistical testing was able to be employed to demonstrate significance. The authors felt this was prudent to stimulate discussion on this topic and objectively quantify what they saw as an increase in total number of cases in their practice. More work needs to be done to calculate the true cost of pandemic and lockdowns on other areas of health other than those directly impacted by hospitalization due to the virus. We have certainly afforded due respect to lethality and cost of a population becoming sick from COVID-19, but other elements improving wellness have fallen by the wayside even though they could be promoted safely.

Opioid overdose deaths have steadily increased by 40% after declining in 2019 during the pandemic in Canada.^24^ As surgeons we often find ourselves the final frontier in helping patients fight infections. Population health measures and medical management of skin infections serve a much larger number of patients than we do. This is a testament to the massive role in reducing morbidity these interventions have and the importance of the accessibility of these services to all during the pandemic. Informed safety measures and policy must direct our practices, especially as it related to the most vulnerable populations during the pandemic. Upper extremity infections are likely the tip of the iceberg of several health issues facing these neglected populations, and we as healthcare providers must be advocates to improve this.

## Supplemental Material

sj-docx-1-han-10.1177_15589447221077377 – Supplemental material for Upper Extremity Infection Related to Intravenous Drug Use: Considering the True Cost of the COVID-19 Pandemic and Lockdown Click here for additional data file. Supplemental material, sj-docx-1-han-10.1177_15589447221077377 for Upper Extremity Infection Related to Intravenous Drug Use: Considering the True Cost of the COVID-19 Pandemic and Lockdown by Matthew McRae, Yaeesh Sardiwalla, Omri Nachmani, Emma Price, Minh Huynh and Christopher Coroneos in HAND
