# Supplementary material for: Upper Extremity Infection Related to Intravenous Drug Use: Considering the True Cost of the COVID-19 Pandemic and Lockdown
Source: Hand (N Y). 2022 Feb 22;18(6):999–1004. doi: 10.1177/15589447221077377 (PMC10470235; doi:10.1177/15589447221077377)
Supplement: sj-docx-1-han-10.1177_15589447221077377 – Supplemental material for Upper Extremity Infection Related to Intravenous Drug Use: Considering the True Cost of the COVID-19 Pandemic and Lockdown [file sj-docx-1-han-10.1177_15589447221077377.docx]

**Appendix A: ICD10 Patient Codes**

L03.0 Cellulitis and acute lymphangitis of finger and toe

L03.01 Cellulitis of finger

L03.011 Cellulitis of right finger

L03.012 Cellulitis of left finger

L03.019 Cellulitis of unspecified finger

L03.02 Acute lymphangitis of finger

L03.021 Acute lymphangitis of right finger

L03.022 Acute lymphangitis of left finger

L03.029 Acute lymphangitis of unspecified finger

L03.1 Cellulitis and acute lymphangitis of other parts of limb

L03.10 Cellulitis and acute lymphangitis of other parts of limb

L03.11 Cellulitis of other parts of limb

L03.111 Cellulitis of right axilla

L03.112 Cellulitis of left axilla

L03.113 Cellulitis of right upper limb

L03.114 Cellulitis of left upper limb

L03.115 Cellulitis of right lower limb

L03.116 Cellulitis of left lower limb

L03.119 Cellulitis of unspecified part of limb

L03.12 Acute lymphangitis of other parts of limb

L03.121 Acute lymphangitis of right axilla

L03.122 Acute lymphangitis of left axilla

L03.123 Acute lymphangitis of right upper limb

L03.124 Acute lymphangitis of left upper limb

L03.125 Acute lymphangitis of right lower limb

L03.126 Acute lymphangitis of left lower limb

L03.129 Acute lymphangitis of unspecified part of limb

L03.9 Cellulitis and acute lymphangitis, unspecified

L03.90 Cellulitis, unspecified

L03.91 Acute lymphangitis, unspecified

L02.413 Cutaneous abscess of right upper limb

L02.414 Cutaneous abscess of left upper limb

L02.419 Cutaneous abscess of limb, unspecified

L02.51 Cutaneous abscess of hand (non-billable)

L02.511 Cutaneous abscess of right hand

L02.512 Cutaneous abscess of left hand

L02.519 Cutaneous abscess of unspecified hand

L02.91 Cutaneous abscess, unspecified

M72.6 Necrotizing fasciitis

M60.01 Infective myositis, shoulder (non-billable)

M60.011 Infective myositis, right shoulder

M60.012 Infective myositis, left shoulder

M60.019 Infective myositis, unspecified shoulder

M60.02 Infective myositis, upper arm (non-billable)

M60.021 Infective myositis, right upper arm

M60.022 Infective myositis, left upper arm

M60.029 Infective myositis, unspecified upper arm

M60.000 Infective myositis, unspecified right arm

M60.001 Infective myositis, unspecified left arm

M60.002 Infective myositis, unspecified arm

M60.041 Infective myositis, right hand

M60.042 Infective myositis, left hand

M60.043 Infective myositis, unspecified hand

M60.04 Infective myositis, hand and fingers (non-billable)

M60.044 Infective myositis, right finger(s)

M60.045 Infective myositis, left finger(s)

M60.046 Infective myositis, unspecified finger(s)

M60.9 Myositis, unspecified

M65.841 Other synovitis and tenosynovitis, right hand

M65.842 Other synovitis and tenosynovitis, left hand

M65.849 Other synovitis and tenosynovitis, unspecified hand

M65.84 M65.841 Other synovitis and tenosynovitis, hand (non-billable)

M00.811 Arthritis due to other bacteria, right shoulder

M00.812 Arthritis due to other bacteria, left shoulder

M00.819 Arthritis due to other bacteria, unspecified shoulder

M00.81 Arthritis due to other bacteria, shoulder (non-billable)

M00.821 Arthritis due to other bacteria, right elbow

M00.822 Arthritis due to other bacteria, left elbow

M00.829 Arthritis due to other bacteria, unspecified elbow

M00.82 Arthritis due to other bacteria, elbow (non-billable)

M00.831 Arthritis due to other bacteria, right wrist

M00.832 Arthritis due to other bacteria, left wrist

M00.839 Arthritis due to other bacteria, unspecified wrist

M00.83 Arthritis due to other bacteria, wrist (non-billable)

M00.841 Arthritis due to other bacteria, right hand

M00.842 Arthritis due to other bacteria, left hand

M00.849 Arthritis due to other bacteria, unspecified hand

M00.84 Arthritis due to other bacteria, hand (non-billable)

A41.0 Sepsis due to Staphylococcus aureus (non-billable)

A41.01 Sepsis due to methicillin susceptible staphylococcus aureus

A41.02 Sepsis due to methicillin resistant staphylococcus aureus

A41.1 Sepsis due to other specified staphylococcus

A41.2 Sepsis due to unspecified staphylococcus
